# Brain Metastases in Sarcomas: A Multicenter Retrospective Cohort Study

**DOI:** 10.3390/cancers16223760

**Published:** 2024-11-07

**Authors:** Ellen Zhang, Sheima Farag, Hilary Dietz, Daniel Wang, Angela Hirbe, Kristen Ganjoo, Brian Van Tine, Shane Zaid, Aisha Miah, Vicki Keedy, Elizabeth Davis, Nam Bui

**Affiliations:** 1 Department of Medicine Division of Oncology, Stanford University School of Medicine, Stanford, CA 94304, USA; ellenz@stanford.edu (E.Z.); kganjoo@stanford.edu (K.G.); 2 Royal Marsden Hospital, London SW3 6JJ, UK; sheima.farag@rmh.nhs.uk (S.F.); shane.zaidi@rmh.nhs.uk (S.Z.); aisha.miah@rmh.nhs.uk (A.M.); 3 Cayuga Health, Ithaca, NY 14850, USA; hilary.dietz@duke.edu; 4 Baylor College of Medicine, Houston, TX 77030, USA; daniel.wang@utsouthwestern.edu; 5 Division of Oncology, Washington University in St. Louis, St. Louis, MO 63130, USA; hirbea@wustl.edu (A.H.); bvantine@wustl.edu (B.V.T.); 6 Division of Hematology/Oncology, Vanderbilt University Medical Center, Nashville, TN 37232, USA; vicki.keedy@vumc.org

**Keywords:** sarcoma, brain metastases, disease prognosis, mortality, incidence, chemotherapy, radiation, surgery

## Abstract

Purpose: Brain metastases (BM) in sarcomas occur rarely and are associated with poor prognosis. This study is a large retrospective cohort describing the demographic and clinical characteristics of these patients, treatment strategies, and survival outcomes. Methods: In total, 81 patients with BM from sarcomas were identified across five sarcoma centers. Demographic data, clinical presentation, and treatment modalities were analyzed. Results: The most common histologies were leiomyosarcoma (12.3%) and undifferentiated pleomorphic sarcoma (12.3%). The median time from sarcoma diagnosis to brain metastases was 1.9 years. Upon presentation, 88.9% of patients with BM from sarcomas were symptomatic with the most common presenting symptom being focal neurological deficits (37.9%) and headaches (22.1%). Higher-grade sarcomas were more likely to metastasize and were usually preceded by metastases to other sites, most commonly the lungs. One-year overall survival was 31% from initial sarcoma diagnosis, and the median time from diagnosis of BM until death was 6.0 months. For treatment, 60 (74.1%) patients had radiation, 39 (48.1%) patients had systemic therapy, and 29 (35.8%) patients had surgery. In a multivariate analysis, surgery (HR 0.30) and chemotherapy (HR 0.23) were found to be significantly correlated with improved survival outcomes. Although radiation as a whole was not found to significantly correlate with survival, improved outcomes were seen with stereotactic radiosurgery (SRS, mOS 11.6 mo) as opposed to whole-brain radiation therapy (WBRT, mOS 8.3 mo). Additionally, patients with leptomeningeal disease were significantly less likely to survive more than one year compared to patients with brain metastases only. Conclusions: Our findings identify that patients with metastatic sarcoma to the brain have poor prognoses, often have concurrent metastasis, and have a median survival of only 6 months. Additionally, our study found that leptomeningeal metastases is a rare presentation with poor survival outcomes. There are various treatment modalities for sarcomas with BM; however, there are no guidelines, unlike in other malignancies. Further research is necessary to evaluate the role of therapeutic measures in terms of type, timing, and outcomes.

## Simple Summary

Brain metastases from sarcomas are uncommon and lead to poor outcomes. This study examines 81 cases of patients with brain metastases from sarcomas to review their symptoms, treatments, and survival. Most patients were symptomatic, often experiencing headaches or focal neurological deficits. Overall, 31% of patients survived one year after their initial sarcoma diagnosis, and the median time from the diagnosis of brain metastasis to death was 6.0 months. Given the rarity and heterogeneity of this disease, there are no structured guidelines for treatment that include chemotherapy, radiation, and surgery. Patients who survived more than two years from the sarcoma diagnosis were significantly more likely to have received chemotherapy, and those who survived more than one year from the brain metastasis diagnosis were significantly more likely to have undergone surgery and chemotherapy. Our findings highlight the urgent need for further research into effective treatment strategies.

## 1. Introduction

Sarcomas are rare tumors that arise in mesenchymal tissues, occurring almost anywhere in the body. Despite their rarity, sarcomas consist of approximately 50 distinct histological subtypes that each have their own unique natural history [1,2]. Sarcomas have a propensity to metastasize to organs including the lung, liver, and bones. The brain is a rare site of metastasis for sarcomas patients with increasing incidence found in patients relapsing after prolonged disease control with treatment [3]. The incidence of brain metastasis in sarcomas is approximately 1–8% with retrospective studies showing that some subtypes, specifically leiomyosarcoma and liposarcoma, and undifferentiated sarcomas, are seen more commonly [4,5,6,7,8,9,10].

In 2020, the Sarcoma-Graded Prognostic Assessment was developed to risk stratify sarcoma patients with brain metastases based on histology, number of brain lesions, and performance status [11]. Specifically, the worst prognosis for histology included adipocytic, smooth muscle, skeletal, chondro-osseous, fibroblastic/myofibroblastic, fibrohistiocytic, “vascular”, and uncertain differentiation tumors [11]. As new therapies emerge and treatments evolve, it is increasingly important to understand how to manage brain metastasis in sarcoma patients [12]. In this study, we analyzed clinical characteristics and treatment patterns of sarcoma patients across multiple institutions who developed brain metastasis.

## 2. Materials and Methods

This retrospective cohort study includes 81 patients diagnosed with sarcomas that metastasized to the brain treated at five sarcoma centers (Stanford Cancer Institute, Vanderbilt University Medical Center, Fred Hutchinson Cancer Center, The Royal Marsden Hospital, Baylor Cancer of Medicine, and Siteman Cancer Center at Washington University) between January 1998 and December 2021. We identified and collected the following data: demographics, characteristics and presentation of brain metastases, and treatment modalities for malignancy.

The following data were collected: age at diagnosis, sex, race, primary tumor characteristics (anatomic location, size, histological type, grade, stage at initial presentation), date and location of first brain metastasis (BM), BM characteristics (location, type, grade, stage, number), symptoms at diagnosis of BM, and treatment prior to and for initial or recurrent BM. All patients were treated at the discretion of their primary oncologist.

Categorical and continuous variables were assessed using percentages and means, with Fisher’s exact test and Student’s *t*-test, respectively. Progression-free survival (PFS) and overall survival (OS) were estimated using the Kaplan–Meier method and compared between groups using the log-rank test. A Cox proportional-hazards model was used for multivariate analysis. All analyses were performed using R version 4.2 and Python version 3.9.

## 3. Results

### 3.1. Characteristics and Presentation of Patients and Primary Sarcoma

A total of 45 men and 36 women were included with the mean age of diagnosis being 43 years and the majority of patients being white (61.7%) (Table 1). At the time of analysis, 58 patients (71.6%) had died of metastatic sarcoma, 16 patients (19.8%) were alive with disease, and 3 patients (3.7%) were alive without evidence of disease. Table 1 provides additional details of sarcoma patients and the characteristics of primary sarcomas. 

The most frequent histologies were leiomyosarcoma (12.3%) and undifferentiated pleomorphic sarcoma (12.3%). The most common primary sarcoma locations were in the extremities (*n* = 41, 50.6%) and trunk (*n* = 25, 30.9%). Characteristics and presentation of brain metastases are detailed in Table 2. While 54 (66.7%) patients had high-grade sarcomas, 5 (6.2%) patients had low-grade sarcoma. Stage at initial presentation of primary sarcoma was localized in 45 (55.6%) and metastatic in 36 (44.4%) patients. The mean (range) primary sarcoma size was 9.9 (0.5 to 28.1) cm. The median (range) time from sarcoma diagnosis to brain metastases was 1.9 (0–34.6) years. Upon presentation, 88.9% of patients with sarcoma and BM were symptomatic; the most common presenting symptom being focal neurological deficits (37.9%) and headaches (22.1%). Most brain metastases were parenchymal (72.8%) rather than leptomeningeal (12.3%) or both (13.6%). Most brain metastases were non-hemorrhagic (56.8%), as opposed to hemorrhagic (13.6%) or both (7.4%). The majority of patients (44.4%) had a single brain metastasis region (Table 3). The majority of patients (70.4%) had their sarcoma metastasize to the lung. Treatment modalities for BM are further detailed in Table 3.

### 3.2. Systemic Therapy

**Figure 1:**
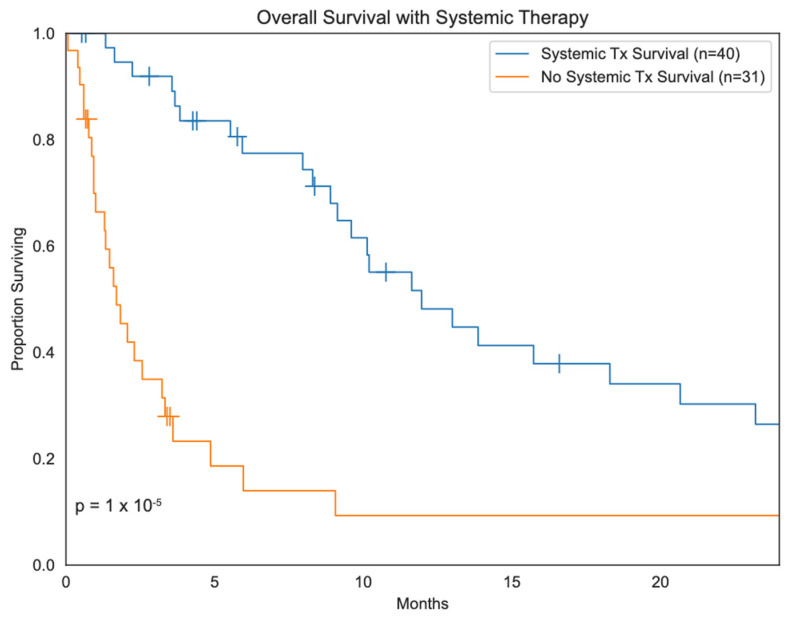
Kaplan–Meier survival analysis of sarcoma patients with brain metastases treated with systemic therapy compared to sarcoma patients with brain metastases not treated with systemic therapy.

In terms of the timing of systemic therapy for sarcoma patients with BM, 7 patients were treated during the adjuvant period, 52 during the metastatic period, and 8 during the neoadjuvant period in relationship to the treatment of the primary sarcoma site. Among the 7 patients who received adjuvant chemotherapy, an anthracycline-based regimen (*n* = 6, 85.7%) and an ifosfamide combination (*n* = 5, 71.4%) were used in the majority of patients. Among the 52 patients treated for BM with chemotherapy, an anthracycline-based regimen was used in 38 patients (73.1%) and an ifosfamide combination in 18 patients (34.6%). Targeted therapy was used in 4 patients specifically with cediranib, pazopanib, an unspecified NOTCH inhibitor, and CAR T-cell therapy. Among 8 patients receiving neoadjuvant therapy, an anthracycline-based regimen (*n* = 8, 100%) and an ifosfamide combination (*n* = 6, 75%) consisted of the majority of treatments. Overall, systemic therapy was a significant predictor of survival (*p* < 0.01) (Figure 1).

Treatment varied widely, with the most common approach being whole-brain radiation therapy (WBRT) alone in 18 patients (22.2%). Additionally, 11 patients (13.6%) underwent systemic therapy combined with WBRT, and another 11 patients (13.6%) received combined surgery, systemic therapy, and stereotactic radiosurgery. 

Brain metastases recurred in 13 patients, and treatment modalities are detailed in Table 4. The majority of patients (53.8%) who had recurrent brain metastases underwent treatment with surgery, systemic therapy, and radiation.

### 3.3. Surgery

**Figure 2:**
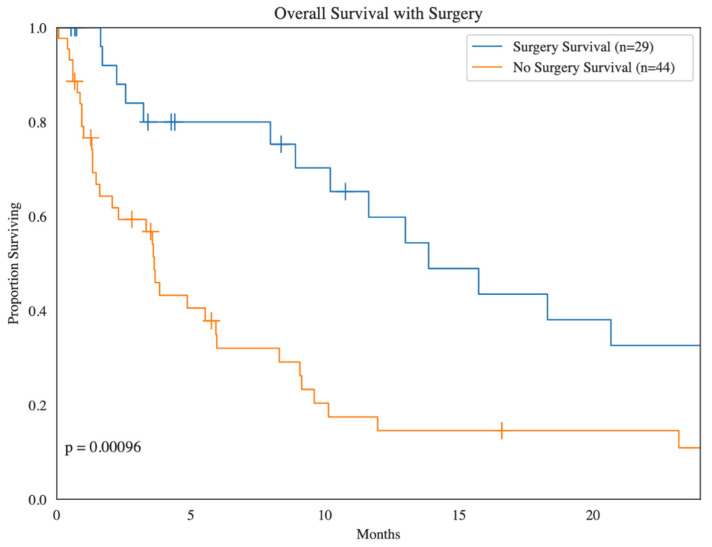
Kaplan–Meier survival analysis of sarcoma patients with brain metastases treated with surgery compared to sarcoma patients with brain metastases not treated with surgery.

Among the 29 patients with surgically resected BM, 16 (55.2%) had localized disease, and 13 (44.8%) had metastatic disease at the time of diagnosis. The average primary tumor size was 9.17 cm, and most patients undergoing surgery had a single brain metastasis (65.5%). Regarding the location of the operated BM, 4 (13.8%) patients had leptomeningeal BM, 20 (67.0%) patients had parenchymal BM, and 5 (17.2%) patients had BM of unknown location. In terms of the type of operated BM, 4 (13.8%) patients had hemorrhagic BM, 16 (55.2%) had non-hemorrhagic BM, and 9 (31.0%) had BM of unknown type. Among those with surgically resected BM, 13 (44.8%) patients had no synchronous extracerebral metastasis while 16 (55.2%) patients had synchronous extracerebral metastases. Of patients with surgically resected BM, 18 (62.1%) patients did not have recurrent BM, 9 (31.0%) patients had recurrent BM, and 2 (6.9%) patients had unknown recurrence of BM. Overall, surgery was a significant predictor of survival (*p* < 0.01) (Figure 2).

### 3.4. Radiation

**Figure 3:**
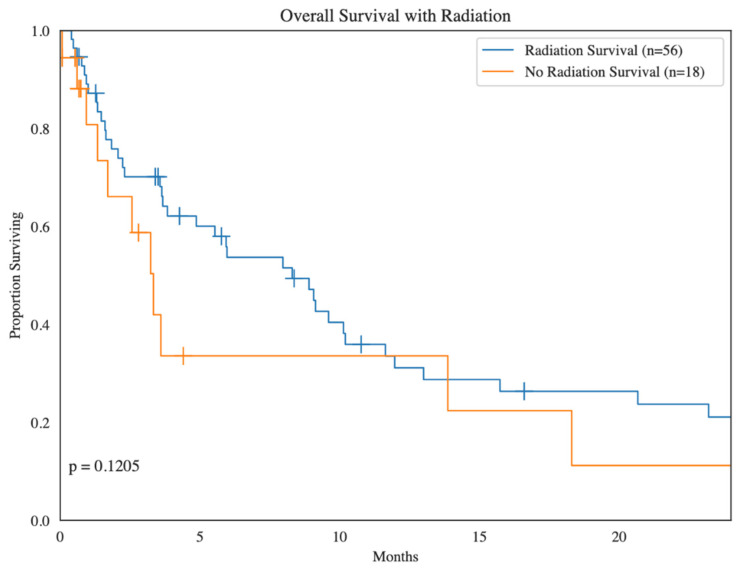
Kaplan–Meier survival analysis of sarcoma patients with brain metastases treated with radiation compared to sarcoma patients with brain metastases not treated with radiation.

Overall, 60 patients underwent radiation for BM: the timing of first radiation was adjuvant in 13 (21.7%) patients, metastatic for 14 (23.3%) patients, neoadjuvant for 4 (6.7%) patients, locally recurrent in 1 (1.7%) patient, and unknown in 28 (46.7%) patients in relation to the treatment of the primary sarcoma site. In terms of the location for radiated BM, 6 (10.0%) patients had leptomeningeal BM, 45 (75.0%) patients had parenchymal BM, 1 (1.7%) patient had both leptomeningeal and parenchymal BM, and 8 (13.3%) patients had BM of unknown location. As for the type of radiated BM, 9 (15.0%) patients had hemorrhagic BM, 36 (60.0%) had non-hemorrhagic BM, 4 (6.7%) had both hemorrhagic and non-hemorrhagic BM, and 11 (18.3%) had BM of an unknown type. Overall, radiation was not a significant predictor of survival (*p* = 0.12) (Figure 3).

**Figure 4:**
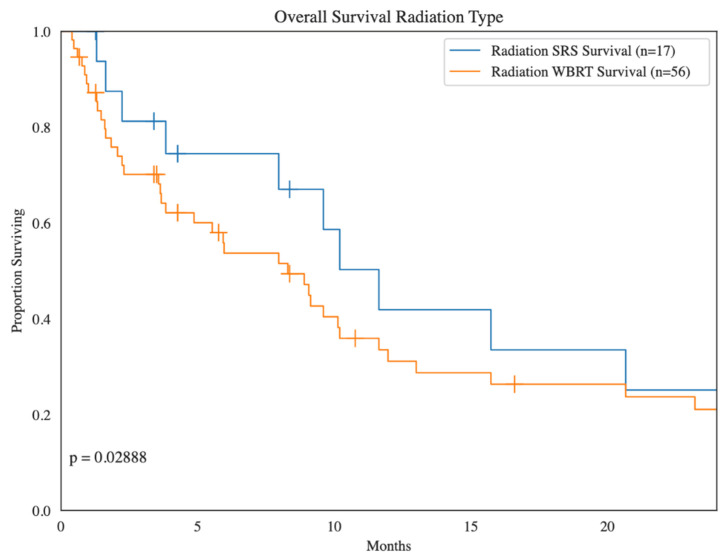
Kaplan–Meier survival analysis of sarcoma patients with brain metastases treated with stereotactic radiosurgery (SRS) compared to sarcoma patients with brain metastases treated with whole-brain radiation therapy (WBRT).

Whole-brain radiotherapy (WBRT) was the most commonly used treatment (48.3%), followed by stereotactic radiosurgery (SRS) (30.0%) and other radiation types (16.7%). Survival among patients treated with SRS was significantly higher compared to those treated with WBRT (Figure 4). Among patients receiving radiation for BM, 40 patients (66.7%) did not have recurrent BM, compared to 11 patients (18.3%) who did (*p* = 0.49).

### 3.5. Overall Survival

**Figure 5:**
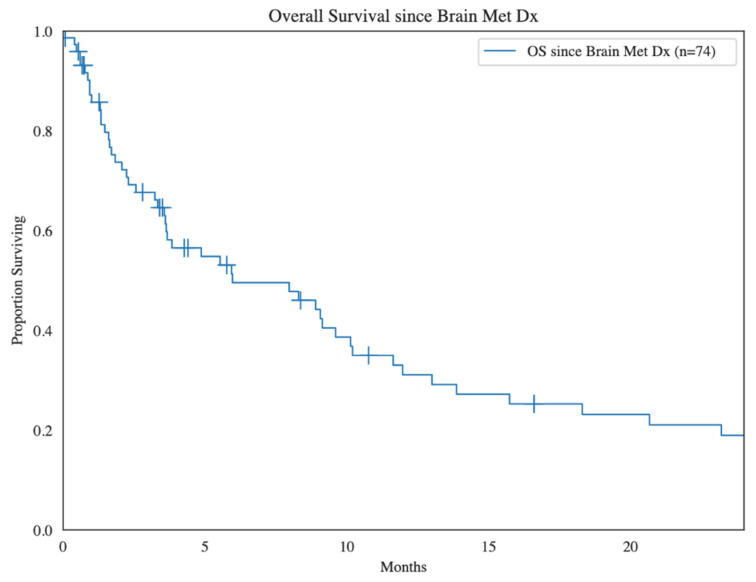
Kapan–Meier survival analysis demonstrating overall survival of sarcoma patients since diagnosis of brain metastasis.

One-year overall survival (OS) in patients with BM was 31% from initial sarcoma diagnosis, and the median (range) time from diagnosis of BM until death was 6.0 (0.5–330.0) months (Figure 5). In multivariate analysis, variables identified as statistically significant for predicting improved survival outcomes included surgery (HR 0.30, *p* < 0.01) and chemotherapy (HR 0.23, *p* < 0.01), but not radiation therapy (HR 0.62, *p* = 0.17). In patients undergoing surgery or chemotherapy, the median survival time after BM diagnosis was markedly improved: 13.9 and 12.0 months, respectively, versus the overall patient median of 6.0 months. For patients undergoing radiation therapy, there was significantly improved survival with stereotactic radiosurgery (SRS, mOS 11.6 mo) as opposed to whole-brain radiation therapy (WBRT, mOS 8.3 mo), likely due to the decreased number of BM found in those necessitating SRS (3.2) compared to WBRT (5.7).

Long-term sarcoma survivors were defined as patients with overall survival greater than two years from initial diagnosis. Approximately 48 (59.3%) patients were long-term sarcoma survivors, with the most common histologies being leiomyosarcoma in 10 (20.8%) patients and undifferentiated pleomorphic sarcoma in 6 (12.5%) patients. The age of long-term sarcoma survivors at diagnosis was comparable (median 42 vs. 43 years old), as was the sex (48% vs. 56% male) of non-long-term sarcoma survivors. Patients with leptomeningeal metastases (12.3%, *n* = 10) compared to those with parenchyma metastases (72.8%, *n* = 59) were significantly more likely to be long-term sarcoma survivors (*p* < 0.01). Long-term sarcoma survivors were significantly more likely to receive chemotherapy (63.8% vs. 33.3%, *p* = 0.01) with no significant difference in receiving radiation (81.3% vs. 65.6%, *p* = 0.12) or surgery (37.5% vs. 34.4%, *p* = 0.82) compared to non-long term BM survivors.

Long-term BM survivors were defined as patients with an overall survival of more than one year from BM diagnosis, comprising 16 (19.8%) patients. Long-term BM survivors most commonly had the following histologies: spindle cell sarcoma (12.5%), undifferentiated pleomorphic sarcoma (12.5%), leiomyosarcoma (12.5%), and alveolar rhabdomyosarcoma (12.5%). The age of long-term BM survivors at diagnosis was significantly younger than that of non-long-term BM survivors (median 32.5 vs. 43.0 years), while the proportion of males was similar in both groups (43.8% vs. 56.0%). Patients with leptomeningeal metastases were not significantly more likely to be long-term BM survivors compared to those with parenchymal metastases. Long-term BM survivors were significantly more likely to receive surgery (68.7% vs. 28.6%, *p* < 0.01) and chemotherapy (87.5% vs. 42.6%, *p* < 0.01), with no significant difference in receiving radiation (81.3% vs. 73.4%, *p* = 0.75) compared to non-long-term BM survivors.

## 4. Discussion

The reported prevalence of BM in cohort studies with patients with sarcoma is less than 1% [8,13]. A large 2002 retrospective study from Memorial Sloan Kettering Cancer Center reviewed 3829 SARCOMA patients and found a prevalence of <1% for brain metastasis, with a 2-year post-metastasis survival of 27% with resection and 0% without resection [8]. Previous studies have found that brain metastases from sarcomas were previously reported to show a slight male predilection that was confirmed in our study with a male-to-female ratio of 1.25:1 [3,6,14,15,16].

The most recent and largest retrospective studies from the MD Anderson Cancer Center (*N* = 112) and French Sarcoma Group (*N* = 246) have provided additional data on the treatment and prognosis of this condition [16,17]. Overall, optimal therapy is not well defined, and prognosis is generally poor, although longer survival in those undergoing surgery [6,14,18]. The incidence of BM from sarcomas has increased in recent years likely due to more aggressive and advanced treatment resulting in prolonged survival in patients with advanced disease [13]. To the best of our knowledge, our report of 81 cases of sarcoma BM patients is by far the largest and most recent multi-site cohort in the literature describing this rare event.

Our study found that primary sarcoma with BM histologies were most commonly leiomyosarcoma (12.3%) and undifferentiated pleomorphic sarcoma (12.3%). This finding is consistent with studies from the MD Anderson Cancer Center and French Sarcoma Group with the most common histologies being undifferentiated sarcoma and leiomyosarcoma, respectively [16,17]. Consistent with previous literature, our results highlight that higher-grade sarcomas were more likely to metastasize and were usually preceded by metastases to other sites, most commonly the lungs [16,17,19].

Our study found that the median time from sarcoma diagnosis to brain metastases was 1.9 years or 23 months. Per the literature, brain metastasis is typically a late event in the natural history of sarcomas, with reported times ranging from 20 to 30 months [3,6,15,17,18]. While there are various treatment modalities, there are no standardized treatments for BM from sarcomas, unlike other malignancies [19,20,21,22,23,24].

Surgery and radiation are often proposed due to poor central nervous system penetration of systemic agents [25]. Our study found that 35.8% of patients underwent surgery and 74.1% of patients received radiation; of those undergoing radiation, 11.7% had SRS and 48.3% had WBRT. Long-term BM survivors were significantly more likely to have undergone both surgery and chemotherapy. 

Meta-analyses of studies have shown that median survival without surgical resection ranges from 1.2 to 2.7 months, while surgery can extend survival to 5.4 to 25.8 months [19]. Our study found that the median survival time for patients who underwent surgery was significantly higher at 13.9 months compared to the overall median survival of 6.0 months from BM diagnosis. Chemotherapy remains the standard of care for advanced or metastatic sarcoma, and our study demonstrated that it significantly improved median survival time [26]. Additionally, chemotherapy was a significant predictor for both long-term sarcoma survivors and long-term BM survivors. In our study, the treatment protocols varied, with common drugs including anthracycline agents (e.g., doxorubicin), ifosfamide, and platinum-based agents (e.g., cisplatin). These treatments have been the mainstay of sarcoma treatment for years [27]. Notably, the efficacy of chemotherapy varies based on histological subtypes, such as osteosarcomas being more chemosensitive compared to clear-cell sarcomas, which are more chemoresistant; additionally, molecular pathology and genotyping are important considerations in chemotherapy [27]. The median survival time of patients who underwent chemotherapy was significantly higher at 12.0 months compared to the overall median survival of 6.0 months from BM diagnosis. Although sarcomas are thought to be relatively radioresistant compared to other tumor types, radiation is often used as a treatment for both therapeutic and palliative control [28,29]. In our study, radiation treatment was not a significant predictor for long-term sarcoma survivors, long-term BM survivors, or brain metastasis recurrence. Hence, consistent with previous studies, we also found prolonged survival with treatment combinations including surgery, chemotherapy, and radiation.

Leptomeningeal metastases are increasingly being recognized as a treatable, yet generally incurable, complication of advanced cancer with treatment options varying widely given their rarity [30,31]. The only literature describing leptomeningeal metastases from sarcomas in a prospective cohort study, published in 2022, found no association between the site of brain metastasis, leptomeningeal, or parenchymal, on disease-specific survival [8]. Our study consisted of 10 patients with leptomeningeal metastases, who when compared to those with parenchymal metastases, were significantly more likely to be long-term sarcoma survivors but not long-term BM survivors. Notably, patients with leptomeningeal metastases were nearly twice as likely to have BM at diagnosis with symptoms. Potentially, these findings might be due to slower progression of leptomeningeal metastases and lower tumor burden in early stages with later detection. Thus, our study is consistent with the literature that the prevalence of leptomeningeal BM from sarcoma is a rare complication.

One of the main strengths of our study is the high number of patients included, which allows us to assess the rare event of sarcoma patients diagnosed with BM. Additionally, we can quantify the primary sarcoma characteristics and the treatment modalities and timelines for managing their malignancy. However, several limitations do exist in our study. One limitation is that some variables for patients were unknown, such as the specificities of treatment regimens and other concurrent metastasis. Another limitation is that sarcomas consist of a variety of histological subtypes, which can often influence treatment decisions, and there are a limited number of patients for each histological subtype. Therefore, a large population-based cohort would be useful in better assessing risk factors and guiding treatment for patients with sarcomas and BM. Thus, our results must be confirmed in future studies.

## 5. Conclusions

We report a cohort of adult sarcoma patients with brain metastasis from several large academic medical centers. Our findings identify that patients with metastatic sarcoma to the brain have poor prognoses often with concurrent metastasis, and a median survival of only 6 months. However, significantly improved outcomes were observed with interventions, suggesting that aggressive treatments are warranted despite the overall poor prognosis. This patient population reveals various modalities for treating sarcomas with BM based on a combination of factors, some of which are addressed in our studies and others that are not investigated or quantifiable. Our study is consistent with previous literature that leptomeningeal metastases are a rare presentation with poor survival outcomes, necessitating more research in this field. In general, sarcoma patients with BM generally have poor prognosis, and the optimal therapeutic approach is not well defined; however, it is evident that combination treatment regimens can improve overall survival. Further research is necessary to evaluate the role of therapeutic measures in terms of type, timing, and outcomes.

## Figures and Tables

**Table 1:** Description of sarcoma patients and primary sarcomas (*N* = 81).

Categories	Subcategories	N (%)
Sex	Male	45 (55.6)
	Female	36 (44.4)
Age of Diagnosis	Mean	44.2
	Median	43
Race	American Indian/Alaska Native	0 (0)
	Asian	4 (4.9)
	Native Hawaiian or Other Pacific Islander	0 (0)
	Black or African American	5 (6.2)
	White	2 (2.5)
	≥2 Race	2 (2.5)
	Unknown/Not Reported	20 (24.7)
Location of Primary	Extremity	41 (50.6)
	Retroperitoneum	5 (6.2)
	Head/Neck	9 (11.1)
	Trunk	25 (30.9)
	Unknown	1 (1.2)
Histological Tyle	Leiomyosarcoma	10 (12.3)
	Undifferentiated Pleomorphic Sarcoma	10 (12.3)
	Alveolar Rhabdomyosarcoma	7 (8.6)
	Angiosarcoma	7 (8.6)
	Myxofibrosarcoma	5 (6.2)
	Ewing Sarcoma	4 (4.9)
	Malignant peripheral nerve sheath tumor	3 (3.7)
	Osteosarcoma	3 (3.7)
	Spindle Cell Sarcoma	3 (3.7)
	Other	29 (35.8)
Grade	Low	5 (6.2)
	High	54 (66.7)
	Unknown	22 (27.2)
Stage at Initial Presentation	Localized	45 (55.6)
	Metastasized	36 (44.4)

**Table 2:** Characteristics and presentation of brain metastasis.

Categories	Subcategories	N (%)
Brain Metastasis Location	Leptomeningeal	10 (12.3)
	Parenchymal Both NA	59 (72.8) 11 (13.6) 1 (1.2)
Type of Brain Metastasis	Hemorrhagic	11 (13.6)
	Non-hemorrhagic	46 (56.8)
	Both	6 (7.4)
	NA	28 (34.6)
Grade	Low	5 (6.2)
	High	54 (66.7)
	NA	22 (27.2)
Synchronous Extracerebral Metastasis	Yes	59 (72.8)
	No	20 (24.7)
	NA	2 (2.5)
Number of Brain Metastases	1 2–4 5–8 >10 NA	36 (44.4) 22 (27.2) 10 (12.3) 12 (14.8) 1 (1.2)
Symptoms from Brain Metastasis	Yes No NA	72 (88.9) 5 (6.2) 4 (4.9)
Symptoms at Diagnosis	Altered mental status	8 (9.9)
	Blurry vision	2 (2.5)
	Dizziness	3 (3.7)
	Drowsy	2 (2.5)
	Fall	2 (2.5)
	Fatigue	1 (1.2)
	Focal neurological deficits Headache	36 (44.4) 21 (25.9)
	Nausea and vomiting	11(13.6)
	Seizure	9 (11.1)

**Table 3:** Treatment modalities for patients with brain metastases.

**Categories**	**Subcategories**	**N (%)**
Surgery of Primary Sarcoma Site Prior to Brain Metastasis	Yes	61 (75.3)
	No NA	19 (23.5) 1 (1.2)
Systemic Therapy Prior to Brain Metastasis	Yes No NA	66 (81.5) 14 (17.3) 1 (1.2)
Treatment of Brain Metastasis	Surgery	29 (35.8)
	Radiation	60 (74.1)
	Systemic therapy	41 (50.6)
	None	8 (9.9)
	NA	1 (1.2)

**Table 4:** Treatment of recurrence of brain metastases.

**Categories**	**Subcategories**	**N (%)**
Recurrence of Brain Metastasis	Yes	13 (16.0)
	No NA	56 (69.1) 12 (14.8)
Treatment of Brain Metastasis Recurrence	Surgery	9 (6.9)
	Systemic therapy	9 (6.9)
	Radiation	11 (8.5)

## Data Availability

The data presented in this study are available on request from the corresponding author.
